# A Comparative Study on Improving Streptozotocin-Induced Type 2 Diabetes in Rats by Hydrosol, Extract and Nanoemulsion Prepared from Cinnamon Leaves

**DOI:** 10.3390/antiox12010029

**Published:** 2022-12-23

**Authors:** Yu-Chi Huang, Bing-Huei Chen

**Affiliations:** 1 Department of Food Science, Fu Jen Catholic University, New Taipei City 24205, Taiwan; jyjariel0324@gmail.com; 2 Department of Nutrition, China Medical University, Taichung 40402, Taiwan

**Keywords:** cinnamon leaves, hydrosol, nanoemulsion, extract, UPLC-MS/MS, type 2 diabetes in rats

## Abstract

*Cinnamomoum osmophloeum* Kanehira (*C. osmophloeum*) contains various biologically active antioxidant compounds such as flavonoids, phenolic acids and cinnamaldehyde. Type 2 diabetes mellitus is a chronic disease of metabolic abnormality caused by insulin deficiency or resistance. The objectives of this study were to analyze various bioactive compounds in *C. osmophloeum* leaves by ultra-high-performance liquid chromatography–tandem mass spectrometry (UPLC-MS/MS), and compare the effects of hydrosol, extract and nanoemulsion prepared from *C. osmophloeum* leaves on improving type 2 diabetes in rats. Our results show that a total of 15 bioactive compounds in *C. osmophloeum* leaves, including quercetin, quercetin-3-*O*-galactoside, quercetin-3-*O*-glucoside, rutin, caffeic acid, benzoic acid, 5-*O*-caffeoylquinic acid, kaempferol 3-β-D-glucopyranoside, *trans*-cinnamic acid, coumarin, cinnamyl alcohol, *p*-coumaric acid, eugenol, kaempferol and cinnamaldehyde, were separated within 14 min for subsequent identification and quantitation by UPLC-MS/MS. The nanoemulsion was successfully prepared by mixing *C. osmophloeum* leaf extract, soybean oil, lecithin, Tween 80 and deionized water in an appropriate proportion with a mean particle size, polydispersity index, zeta potential and encapsulation efficiency of 36.58 nm, 0.222, −42.6 mV and 91.22%, respectively, while a high storage and heating stability was obtained. The animal experiment results reveal that the high-dose nanoemulsion was the most effective in reducing both fasting blood glucose and oral glucose tolerance test value, followed by low-dose nanoemulsion, high-dose extract, low-dose extract and leaf powder in hydrosol. A similar trend was shown in reducing serum insulin and the homeostatic model assessment of insulin resistance index. In addition, the contents of serum biochemical parameters, including total cholesterol, triglyceride, aspartate aminotransferase, alanine aminotransferase, uric acid, urea nitrogen and creatinine, were reduced, with the high-dose nanoemulsion showing the most pronounced effect. Collectively, the high-dose nanoemulsion may possess great potential to be developed into a hypoglycemic health food or botanic drug.

## 1. Introduction

*Cinnamomum osmophloeum* kanehira (*C. osmophloeum*), commonly known as Taiwan native cinnamomum or pseudocinnamomum, is an endemic plant in Taiwan. Classically, it belongs to the *Lauraceae* family and the *Cinnamomum* genus. Because of its unique cinnamon aroma, *C. osmophloeum* leaves are often used as raw material for the production of food additives, spices and Chinese herbal medicines [1]. Of the various bioactive compounds, the dominant one in *C. osmophloeum* leaves is cinnamaldehyde, whose content accounts for 70% [2] or 88.5% [3], depending on species, growth location and environment. In addition, *C. osmophloeum* leaves are shown to contain a significant level of flavonoids and phenolic acids [4]. However, two species, *Cinnamomum cassia* Blume and *Cinnamomum zeylanicu* Brey, are known to contain high levels of coumarin, a toxic compound proven to be hepatotoxic and carcinogenic. Conversely, for the cinnamon leaves grown in Taiwan, only a trace amount of coumarin is present, and should possess greater safety than those imported from foreign countries [5]. In recent years, many studies dealing with chemical composition and pharmacological activities of cinnamons have been published, illustrating that the intake of cinnamon and its related products may be effective in the treatment of chronic diseases such as diabetes [6] and cancer [7].

The use of herbs/spices as a source of antioxidants in traditional medicine for the treatment of diabetes has been well documented [8]. More specifically, cinnamon, being a rich source of antioxidants, including cinnamaldehyde, flavonoids and phenolic acids, has been demonstrated to possess excellent antioxidant activity, with molecular mechanisms involving the reduction in oxidative stress and elevation of the activities of antioxidant enzymes such as superoxide dismutase (SOD), glutathione S-transferase (GST), catalase, glutathione peroxidase, heme oxygenase-1, NADPH dehydrogenase and quinone 1 [9]. It has a long history of medicinal use worldwide, and of the various cinnamon species, *Cinnamomum osmophloeum*, an endemic tree native of Taiwan, has been shown to exhibit several biological activities, including anti-oxidation, anti-inflammation, anti-cancer and anti-diabetes [10,11,12,13].

Nanotechnology refers to the technology dealing with the fabrication and application of nanomaterials with sizes from 1–100 nm; the physical and chemical properties of a substance will vary with this size, while its bioavailability and physiological activities can be affected substantially. Specifically, the water solubility, stability and bioactivity of nanomaterials in vivo can be greatly enhanced through encapsulation, and thus the drug dosage can be reduced with minimum side effects. More importantly, through the preparation of nanoemulsions or microemulsions, it is possible that chronic diseases such as diabetes can be treated through passive targeting [14]. Moreover, through the modification of the surface of nanodrugs or bioactive nanocompounds for conjugation with ligands, the possible treatment of cancer can be achieved through active targeting [15].

According to the World Health Organization [16], diabetes mellitus is defined as a chronic abnormal metabolic disease. Accordingly, diabetes mellitus can be divided into types 1 and 2, with the former caused by the abnormal function of the immune system, leading to the destruction of β cells in islets and impairment of insulin secretion, while the latter is caused by insulin resistance and inadequate insulin secretion due to poor living habit and obesity [17]. The β cells in pancreatic islets are vital in maintaining normal glucose levels in the body, as they can secret insulin to facilitate glucose uptake by the recipient organs such as the brain, liver, muscle and adipose tissue. Of the various risk factors, obesity is key for type 2 diabetes to occur, as it can desensitize glucose recipient organs towards insulin action [18]. The metabolic disorder of nutrients such as carbohydrates, proteins and lipids can thus occur, making it difficult for cells to utilize carbohydrates and thus showing hyperglycemia symptoms. Consequently, long-term hyperglycemia can damage body tissues and organs, especially the nerves and blood vessels, resulting in the occurrence of many complications such as cardiovascular disease, bone fracture, and malfunctions of the kidney, retina and nerves [19].

According to the latest statistics report from the International Diabetes Federation [20], the number of people with diabetes worldwide has exceeded 500 million, and it is estimated that by 2030, a total of 643 million people, accounting for 11.3% of the total population, will suffer from diabetes, and this number is expected to jump to 783 million by 2045. Based on a report by the Taiwan Food and Drug Administration [21], the proportion of adults suffering from diabetes in Taiwan accounts for 11% of the total population, representing about 2.3 million diabetic patients, while new diabetic cases of about 160,000 occur every year. Thus, it is imperative to find effective medicine for the treatment of diabetic patients without side effects. The objectives of this study were to analyze the contents of cinnamaldehyde and the other bioactive antioxidant compounds in *C. osmophloeum* leaves by employing an UPLC-MS/MS technique for the subsequent preparation of *C. osmophloeum* leaf extract, nanoemulsion and hydrosol, in order to study their anti-diabetic effects using an animal model.

## 2. Materials and Methods

### 2.1. Reagents and Instrumentation

All the reagents and instruments used in this study, along with their vendor/manufacturer details, are provided in the Supplementary Materials.

### 2.2. Processing of C. osmophloeum Leaves and Hydrosol

The *C. osmophloeum* leaves used in this experiment were harvested in Pinlin district, New Taipei City, Taiwan, in December, and provided by Tou-Fu Co (Taipei, Taiwan). After transportation to our laboratory, the leaves were washed first and then dried at room temperature for 1 day, after which the leaves were subjected to freeze drying, oven drying and baking separately. The freeze drying condition was controlled at −45 °C, vacuum degree of 125 mT and drying time of 4 days, while the oven drying condition was at 60 °C for 2 h and baking was at 60 °C for 3 h. In addition, a total of about 30 L of hydrosol was obtained by distilling 8 kg of *C. osmophloeum* leaves with 50 L of pure water at 100 °C for 3 h, and the residual leaves were also collected.

### 2.3. Determination of Basic Composition in C. osmophloeum Leaves

The basic components in *C. osmophloeum* leaves, including moisture, crude fat and crude protein content, were determined using the methods of the National Standards of the Republic of China (CNS) [22], while the ash content was established using the method of CNS [23].

### 2.4. Effect of Solvent Variety on Extraction Efficiency of Total Phenolic Acid and Total Flavonoid content in C. osmophloeum Leaves, Hydrosol and Residual C. osmophloeum Leaves

The effect of different extraction solvents, including 30%, 50%, 70% ethanol solutions and 100% H_2_O, on the contents of total phenolic acids and total flavonoids in *C. osmophloeum* leaves and by-products were evaluated. A method based on the of Kao et al. [24] was modified to determine total phenolic acids and total flavonoids in *C. osmophloeum* leaves. For total phenolic acid determination, a 50 µL extract of *C. osmophloeum* leaves was collected and mixed with 200 µL of Folin–Ciocalteu phenol reagent, after which the mixture was reacted in the dark for 5 min, followed by the addition of 1 mL of sodium carbonate solution (15%), mixing and reacting in the dark for 1 h, and the absorbance was measured at 750 nm. Then, the content of total phenolic acids (expressed as gallic acid equivalent) was calculated based on the standard curve of gallic acid solution (in ethanol), which was obtained by plotting five different concentrations (50, 100, 200, 300 and 400 µg/mL) of gallic acid standard separately against absorbance. Likewise, for total flavonoid determination, a 200 µL extract of *C. osmophloeum* leaves was collected and mixed with 30 µL of sodium nitrite solution (5%), after which the mixture was stood at room temperature for 5 min. Then, 60 µL of aluminum chloride solution (10%) was added, followed by standing at room temperature for 5 min, adding 300 µL of sodium hydroxide solution (1 M) and 200 µL of chloroform, centrifuging, collecting the supernatant and measuring absorbance at 510 nm. The content of total flavonoids (expressed as quercetin equivalent) was calculated based on the standard curve of quercetin, which was obtained by plotting six different concentrations of quercetin (5, 10, 25, 50, 100 and 200 µg/mL) separately against absorbance.

Residual *C. osmophloeum* leaves were subjected to analysis of total phenolic acid and total flavonoid content using the same approach as shown above with 30% ethanol as the extraction solvent. However, for hydrosol, mainly composed of 97% water, it was directly analyzed without solvent extraction.

### 2.5. Extraction and Analysis of Cinnamaldehyde and the Other Bioactive Compounds in C. osmophloeum Leaves and Hydrosol by UPLC-MS/MS

Both methods of Eidi et al. [25] and Wardatun et al. [26] were modified to evaluate the extraction efficiency of cinnamaldehyde in *C. osmophloeum* leaves. Initially, 40 mL of 30% or 80% ethanol in water was mixed separately with 1 g of *C. osmophloeum* leaves, after which the mixture was sonicated at 60 °C for 2 h and centrifuged at 4000 rpm for 20 min (25 °C). Then, the supernatant was collected, evaporated to dryness, dissolved in 10 mL of ethanol and filtered through a 0.22 µm membrane filter for subsequent UPLC-MS/MS analysis. However, for hydrosol, it was directly analyzed by UPLC-MS/MS without solvent extraction.

For UPLC-MS/MS analysis, a Luna Omega C18 column (100 mm × 2.1 mm ID, particle size 1.6 μm) and a gradient mobile phase of 0.025% acetic acid solution (A) and methanol containing 0.025% acetic acid (B) was used with the initial ratio of 83% A and 17% B and increased to 20% B in 1 min, 40% B in 5 min, 55% B in 10 min and 99% B in 14 min. A total of 15 bioactive compounds, including quercetin, quercetin-3-*O*-galactoside, quercetin-3-*O*-glucoside, rutin, caffeic acid, benzoic acid, 5-*O*-caffeoylquinic acid, kaempferol 3-β-D-glucopyranoside, trans-cinnamic acid, coumarin, cinnamyl alcohol, p-coumaric acid, eugenol, kaempferol and cinnamaldehyde, were separated within 14 min with flow rate at 0.3 mL/min and column temperature at 30 °C. A tandem mass spectrometer with multiple reaction monitoring (MRM) mode was used for detection in both ESI positive and negative ion modes with desolvation gas flow at 800 L/h, cone gas flow at 150 L/h, nebulizer pressure at 7 bar, vaporizer temperature at 200 °C, capillary voltage at 3000 V (positive) and 2500 V (negative), cone voltage at 30 V and source offset voltage at 30 V. The various bioactive compounds in *C. osmophloeum* leaves were then identified by comparing retention time and mass spectra of standards with those of unknown peaks.

### 2.6. Method Validation

The repeatability (intra-day variability) analysis was performed by determining various bioactive compound contents in *C. osmophloeum* leaves in the morning, afternoon and evening on the same day, with three replicates each for a total of nine replicates, while the determination of intermediate precision (inter-day variability) was carried out by analyzing various bioactive compound contents in *C. osmophloeum* leaves in the morning, afternoon and evening, each on the first, second and third day for a total of nine analyses. For the recovery study, 0.1 and 1 µg each of quercetin, coumarin, quercetin-3-*O*-galactoside, quercetin-3-*O*-glucoside, rutin, p-coumaric acid and 5-*O*-caffeoylquinic acid standards; 1 and 10 µg each of caffeic acid, benzoic acid, kaempferol 3-β-D-glucopyranoside and kaempferol standards; 10 and 100 µg each of *trans*-cinnamic acid, cinnamyl alcohol and eugenol standards; as well as 1000 µg and 10,000 µg of the cinnamaldehyde standard were added to 0.2 g of *C. osmophloeum* leaves for extraction and UPLC-MS/MS analysis. Following quantitation, the recovery data of each compound were calculated based on the relative ratio of each compound content after UPLC-MS/MS analysis to that before UPLC-MS/MS analysis. Additionally, a series of the standard concentrations, including 0.1, 0.5, 1, 2, 4, 6, 10, 20, 30, 50 and 100 ng/mL, were prepared separately and injected into UPLC-MS/MS three times for the determination of the limit of detection (LOD) based on S/N ≥ 3 and the limit of quantitation (LOQ) based on 3.3 × LOD.

### 2.7. Quantitation of Cinnamaldehyde and the Other Bioactive Compounds in C. osmophloeum Leaves and Hydrosol

The standard curves of quercetin, quercetin-3-*O*-galactoside, quercetin-3-*O*-glucoside, rutin, caffeic acid, benzoic acid, 5-*O*-caffeoylquinic acid, kaempferol 3-β-D-glucopyranoside, *trans*-cinnamic acid, coumarin, cinnamyl alcohol, *p*-coumaric acid, eugenol, kaempferol and cinnamaldehyde were each prepared with eight concentrations of 10, 20, 50, 100, 200, 300, 400 and 500 ng/g separately, and analyzed by UPLC-MS/MS in triplicate. Then, the standard curve of each compound was drawn by plotting concentration against peak area, and both the linear regression equation and coefficient of determination (R^2^) of each standard curve were obtained. Following UPLC-MS/MS, the contents (ng/g) of cinnamaldehyde and the other bioactive compounds in *C. osmophloeum* leaves were calculated using the formula, (Y-b/a)×DF, where DF is the dilution factor (10,000 for cinnamaldehyde and 100 for all the other bioactive compounds) and Y is the peak area of bioactive compounds, while a and b are the slope and intercept of the linear regression equation, respectively.

### 2.8. Preparation of C. osmophloeum Leaf Nanoemulsion

The *C. osmophloeum* leaf nanoemulsion (10 mL) containing 10,000 ppm cinnamaldehyde was prepared by mixing 1% soybean oil (0.1 g), 5% Tween 80 (0.5 g), 2% lecithin (0.2 g) and 1% PEG (0.1 g) in a round bottom bottle, followed by dissolving in 99% ethanol and mixing thoroughly. Then, the *C. osmophloeum* leaf extract (20 mL) was added and concentrated under reduced pressure to remove solvent, while a thin film was formed in the bottle. Next, 91% deionized water (9.1 g) was added and shaken in an ultrasonic shaker for 30 min, and a dark green appearance of *C. osmophloeum* leaf nanoemulsion was prepared.

### 2.9. Determination of C. osmophloeum Leaf Nanoemulsion Characteristics

The zeta potential of *C. osmophloeum* leaf nanoemulsion was analyzed by collecting a portion (100 µL) and then diluting it 200 times with deionized water for determination at 25 °C via a zeta potential analyzer. The particle size and polydispersity index (PDI) of *C. osmophloeum* leaf nanoemulsion were analyzed by collecting a portion (100 µL) and diluting 200 times with 25 mM of dihydrogen potassium phosphate buffer solution (pH 5.3–5.5). Then, this solution was filtered through a 0.45 µm membrane filter and poured into a polystyrene colorimetric tube for determination of particle size and PDI by a dynamic light scattering analyzer (DLS). Additionally, the particle size and shape of *C. osmophloeum* leaf nanoemulsion were analyzed by diluting 50 times with deionized water, and a portion (100 µL) was collected and dropped onto a carbon-coated copper grid for 90 s. Then, the excess sample was removed with filter paper for negative staining with 20 µL of phosphotungstic acid (2%) for 2 min, followed by removing the excess sample again with filter paper and drying overnight in an oven for determination using a transmission electron microscope (TEM).

The encapsulation efficiency of cinnamaldehyde in *C. osmophloeum* nanoemulsion was estimated by determining the free cinnamaldehyde, followed by subtracting it from the total cinnamaldehyde and expressing it as a percentage. Initially, free cinnamaldehyde was obtained by mixing n-hexane (400 μL) and *C. osmophloeum* nanoemulsion (100 μL) and shaking slightly to dissolve the free cinnamaldehyde in the supernatant n-hexane phase, while the total cinnamaldehyde was obtained by mixing the nanoemulsion (100 μL) with 99% ethanol (400 μL), followed by ultrasonic vibration for 2 h to release the total cinnamaldehyde, which was subsequently analyzed by HPLC with UV detection at 280 nm.

For the stability study, the *C. osmophloeum* leaf nanoemulsion was stored at 4 °C for 3 months, during which time a portion was collected every 15 days for the determination of particle size and PDI by DLS, as well as zeta potential by a zeta potential analyzer. Similarly, a sample of *C. osmophloeum* leaf nanoemulsion (200 µL) was collected in a tube and placed into a water bath for heating at 40, 70 and 100 °C for 0.5, 1, 1.5 and 2 h, and the particle size, PDI and zeta potential were determined by DLS after heating.

### 2.10. Animal Experiment

A total of 56 6-week-old Wistar male rats were procured from Taiwan BioLASCO Co (Taipei, Taiwan), and these animals were housed in individual ventilation cages with the temperature at 21 ± 2 °C and relative humidity at 55 ± 10% for 12 h under light in Fu Jen University Animal Center. This animal experiment was approved by Fu Jen University Animal Care and Use Committee (permission no. A11044), and the methods dealing with animal experiments were carried out based on approved guidelines [21]. All the rats were fed with a laboratory rodent diet (LabDiet Co, St Louis, MO, USA) and water ad libitum, while the body weight, food and water intake of each rat were measured every week for a total of 5 weeks.

A method based on a report by the TFDA [27] was used to induce diabetes mellitus in rats. When the rats were 7 weeks old, they were ready to induce hyperglycemia. Before induction, nicotinamide (NA) and streptozotocin (STZ) were dissolved separately in physiological saline containing 0.1 M sodium citrate buffer (pH 4.5), and the rats were fasted for 12 h. Then, NA was injected at a dose of 230 mg/kg intraperitoneally, followed by the injection of STZ at 65 mg/kg 15 min later. The normal control group was injected with saline and sodium citrate buffer solution intraperitoneally. On the 7th day of induction, blood was collected from the tail vein to measure the overnight fasting blood glucose. If the fasting blood glucose (FBG) was >200 mg/dl, the above procedures were deemed to have induced diabetes in rats successfully, and a high-fat diet containing 60% fat was started for 4 weeks. Based on two reports by Bisht and Sisodia [28] and Kumar et al. [29], the recommended dose for cinnamaldehyde is 20 mg/kg, and thus a total of seven groups with eight rats each were used as follows: (1) normal control (NC), (2) diabetic control (DC) fed with deionized water, (3) diabetic rats with cinnamon powder in hydrosol (0.5 g/10 mL) with a dose of 10 mL/kg (HP), (4) diabetic rats with low-dose extract at 20 mg/kg (EL), (5) diabetic rats with low-dose nanoemulsion at 20 mg/kg (NL), (6) diabetic rats with high-dose extract at 60 mg/kg (EH), and (7) diabetic rats with high-dose nanoemulsion at 60 mg/kg (NH).

### 2.11. Biochemical Parameter Determination

The FBG level was measured using a glucometer (GE100, New York, NY, USA). Following 12 h fasting, rats were fixed with a rat holder. Then, the rat tails were warmed for 2–3 min with a thermal pad for blood collection from the tail vein for measuring FBG once every week. The oral glucose tolerance test (OGTT) was conducted on the 4th week. Following the administration of fasting rats with their respective treatments for 30 min on the 4th week, glucose (1 g/kg) was provided by tube feeding, and then the blood glucose level was determined before giving sugar and after giving sugar to the rats at 30, 60 and 120 min intervals. Serum aspartate aminotransferase (AST), alanine aminotransferase (ALT), alkaline phosphatase (ALP), total cholesterol (TC), triglycerides (TG), creatinine (CREA), blood urea nitrogen (BUN) and uric acid (UA) were determined using a VetTest automated clinical chemistry analyzer. Serum insulin was analyzed using a specific antibody radioimmunoassay kit, and the homeostatic model assessment of insulin resistance (HOMA-IR) was calculated using the following formula: fasting blood glucose (mmol/L) x fasting insulin concentration (µU/mL).

### 2.12. Statistical Analysis

All the data were subjected to statistical analysis using statistical analysis system (SAS) software, version 6 (Cary, NC, USA) [30]. Additionally, analysis of variance (ANOVA) was conducted to test for significance in mean comparisons (*p* < 0.05), followed by Duncan’s multiple range test.

## 3. Results and Discussion

### 3.1. The Basic Composition of C. osmophloeum Leaves

Table 1 shows the basic composition of *C. osmophloeum* leaves before and after drying. Evidently, compared to fresh leaves, the percentage of moisture content substantially decreased in dried leaves. This result is similar to that presented in a report by Chen [31], showing that the moisture content was 55.15 ± 2.65% in fresh leaves and reduced to 7.36 ± 0.14% after drying. However, the percentages of ash, crude protein and crude fat increased after drying, with their contents being about twofold higher than those in fresh cinnamon leaves.

### 3.2. Total Phenolic Acids and Total Flavonoids in C. osmophloeum Leaves and Hydrosol

The effect of water and different proportions of ethanol on the contents of total phenolic acids and total flavonoids in *C. osmophloeum* leaves is shown in Table 2. The highest content of total phenolic acid was shown for 30% ethanol, followed by 50% ethanol, 70% ethanol and 100% water. A similar trend was found for total flavonoid, with the highest content being shown for 30% ethanol, followed by 50% ethanol, 100% water and 70% ethanol. By comparison, 30% ethanol generated the highest yield of both total phenolic acids and total flavonoids from *C. osmophloeum* leaves, and thus was used as the extraction solvent for subsequent experiments. However, for the analysis of cinnamaldehyde and the other bioactive compounds in cinnamon leaves by UPLC-MS/MS, 80% ethanol was selected as the extraction solvent, as a much higher yield of cinnamaldehyde was shown.

Table 3 shows the contents of total phenolic acids and total flavonoids in *C. osmophloeum* leaves as affected by different drying treatments. Compared to fresh leaves, the total phenolic acid and flavonoid content in *C. osmophloeum* leaves increased after freeze drying, oven drying and baking, with no significant difference (*p* > 0.05) being found among these treatments. However, for subsequent experiments, the oven drying method was chosen because of its low cost and short drying time. In several previous studies, the contents of total phenolic acids and total flavonoids in different species of cinnamon leaves were reported to be from 0.69–2.70 mg/g and 0.90–2.73 mg/g, respectively [32]. In cinnamon barks of different tree age, the contents of total phenolic acids and total flavonoids were from 37.27–376.35 mg/g and 9.12–102.80 mg/g, respectively [33]. Yang et al. [34] further reported that cinnamon bark contained a higher level of total phenolic acids (95.3 mg/g) than cinnamon leaves (88.5 mg/g), while the cinnamon leaves contained a higher level of total flavonoids (33.4 mg/g) than cinnamon bark (20.3 mg/g). Thus, by taking both phenolic acid and flavonoid content into account, cinnamon leaves can still be a rich source of phenolic acid and flavonoid. 

In addition, during the distillation of cinnamon leaves, the major product, hydrosol, was obtained. Only low total phenolic acid (0.04 mg/mL) and total flavonoid (0.03 mg/mL) contents were present in hydrosol. This outcome indicates that most phenolic acids and flavonoids were present in residual leaves, with the total content being 15.01 and 15.09 mg/g, respectively. In a similar study, Li et al. [35] also reported the presence of total phenolic acid at 0.002 to 0.04 mg/mL in hydrosol.

### 3.3. UPLC-MS/MS Analysis of Cinnamaldehyde and the Other Bioactive Compounds in C. osmophloeum Leaves and Hydrosol

**Figure 1:**
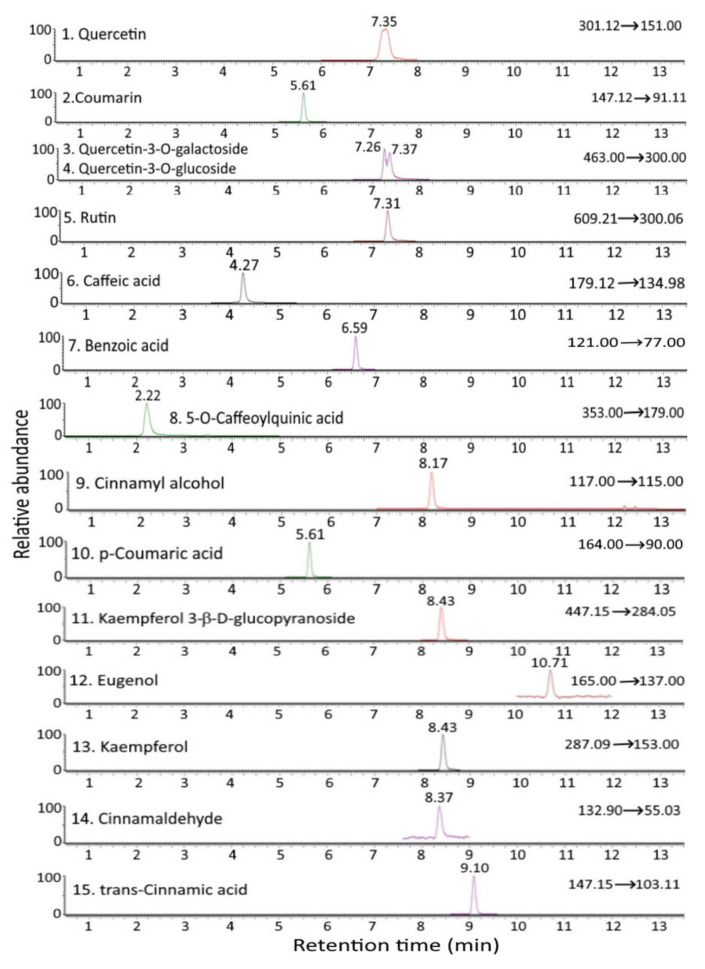
UPLC-MS/MS chromatograms of standards of cinnamaldehyde and various bioactive compounds as detected by MRM mode.

**Figure 2:**
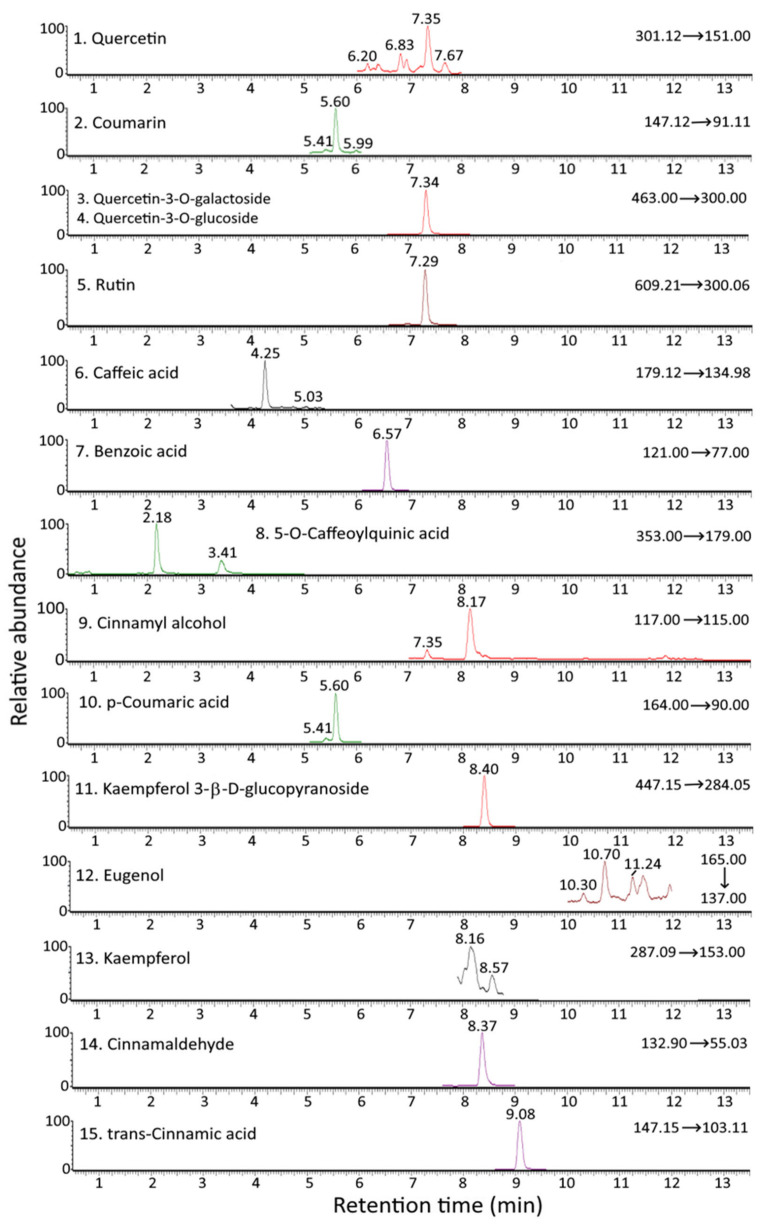
UPLC-MS/MS chromatograms of cinnamaldehyde and various bioactive compounds in *C. osmophloeum* leaves as detected by MRM mode.

Figure 1 and Figure 2 show the UPLC-MS/MS chromatograms of standards and various bioactive compounds in *C. osmophloeum* leaves, respectively. A total of 15 compounds, as shown in Table 4, were separated within 14 min, however, both quercetin-3-*O*-galactoside and quercetin-3-*O*-glucoside overlapped. The identification of all 15 bioactive compounds in *C. osmophloeum* leaves and hydrosol was conducted by comparing retention times and mass spectra of unknown peaks with those of standards and those reported in the literature [36,37,38,39].

### 3.4. Method Validation

Table 5 shows the quality control data of cinnamaldehyde and the other bioactive compounds in *C. osmophloeum* leaves as determined by UPLC-MS/MS. The LOD of all 15 compounds ranged from 0.08–8.40 ng/g, and the LOQ from 0.24–25.19 ng/g, while the relative standard deviation (RSD) of the intra-day variability and inter-day variability ranged from 1.93–8.21% and 1.81–8.93%, respectively (Table 5). In addition, the high accuracy of this UPLC-MS/MS method is shown in Table 6, as evidenced by the high recoveries (90.21–106.62%) of cinnamaldehyde and the other bioactive compounds. Apparently, the RSDs of intra-day/inter-day variability and recovery values meet the requirement set by the TFDA [40], implying that high precision and accuracy was attained for the UPLC-MS/MS method developed in this study.

### 3.5. Quantitation of Cinnamaldehyde and the Other Bioactive Compounds in C. osmophloeum Leaves and Hydrosol

The linear regression equations obtained from the calibration curves showed a coefficient of determination (R^2^) > 0.98 for all 15 compounds (Table S1), and were used for their quantitation in *C. osmophloeum* leaves. The quantitation data of cinnamaldehyde and the other bioactive compounds in *C. osmophloeum* leaves and hydrosol are also shown in Table 4, with their levels in the former ranging from 0.56–5240 μg/g, while only benzoic acid, cinnamyl alcohol, eugenol, cinnamaldehyde and trans-cinnamic acid were detected in hydrosol, which ranged from 0.39–1435 μg/g. Apparently, cinnamaldehyde is the dominant compound in both *C. osmophloeum* leaves and hydrosol. Furthermore, compared to 30% ethanol, a much higher content of cinnamaldehyde in cinnamon leaves was found with 80% ethanol as the extraction solvent (Table 4).

Similar outcomes were reported in several previous studies. For instance, Fang et al. [41] reported the presence of cinnamaldehyde at 420–23,790 µg/g in cinnamon leaves of different tree age, in which the leaves of 3-year-old trees showed the highest content. Yeh et al. [5] analyzed cinnamaldehyde content in cinnamon leaves grown in different locations, and a level of 8900–26,100 µg/g was shown. Ding et al. [42] further determined bioactive compounds in 56 species of cinnamon barks and branches from different locations; cinnamaldehyde was shown to be present at the highest level (86,250 μg/g), followed by eugenol (14,400 μg/g), coumarin (5790 μg/g), cinnamyl alcohol (1130 µg/g) and cinnamic acid (870 µg/g). Comparatively, the cinnamaldehyde content in cinnamon leaves shown in our study was lower than in most published reports, which can be attributed to the difference in species, growth location, environment and analytical methods used. Nevertheless, we have to point out that only a trace amount of coumarin (2.46 μg/g) was present in dried cinnamon leaves investigated in our study, which should greatly enhance the safety of *C. osmophloeum* leaves grown in Taiwan. As coumarin is a toxic compound, its content in beverages has to be controlled and maintained at <2.0 mg/kg [43]. Based on a report by the German Federal Institute for Risk Assessment, the daily intake of coumarin for a 60 kg adult should be <6 mg [44]. Thus, as long as the daily intake of cinnamon powder is controlled at <2.5 kg, the acceptable daily intake cannot be exceeded. Wang et al. [2] investigated the coumarin content in different species of cinnamon powder, including *C. cassia*, *C. loureiroi* and *C. burmannii*, and a range of 5–9300 µg/g was reported.

### 3.6. Preparation and Characterization of C. osmophloeum Leaf Nanoemulsion

**Figure 3:**
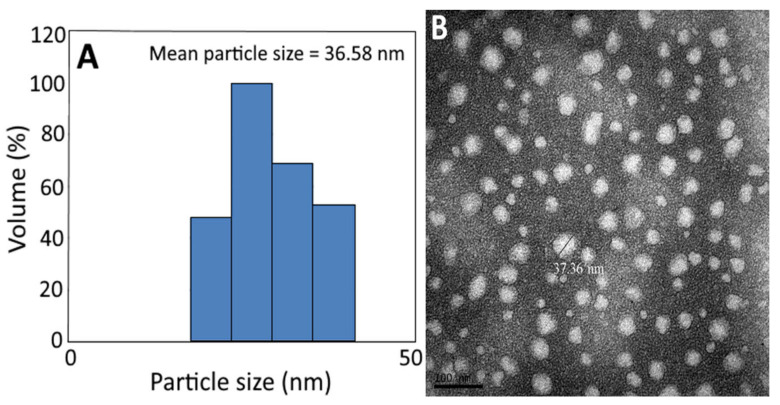
Particle size distribution of *C. osmophloeum* leaf nanoemulsion, as determined by the dynamic light scattering (DLS) method, with a mean particle size of 36.58 nm (**A**), along with its transmission electron microscopy image, with a mean particle size of 37.36 nm (**B**).

The average particle size (36.58 nm) and PDI (0.222) of *C. osmophloeum* nanoemulsion, as determined by DLS (Figure 3A and Table 7), implied that an even distribution of particles in this nanoemulsion was attained, as it was reported that the PDI should be controlled between 0.1 and 0.3 to obtain a narrow distribution [45]. Similarly, the mean particle size of *C. osmophloeum* nanoemulsion, as determined by TEM, was found to be 37.36 nm, with a round shape (Figure 3B).

In several previous studies, Zhang et al. [46] prepared a nanoemulsion composed of cinnamon essential oil, Tween 80, ethanol and deionized water; the average particle size and PDI were shown to be 8.69 nm and 0.22, respectively. In another study, a nanoemulsion containing cinnamaldehyde, Tween 80 and deionized water was prepared, with the average particle size and PDI being 55.5 nm and 0.08, respectively [47]. Similarly, the average particle size of a nanoemulsion containing cinnamon essential oil, coconut oil, Tween 80 and deionized water was 100 nm [48], while a larger particle size of 185 nm with a PDI of 0.22 was shown in a nanoemulsion composed of whey protein isolate, glucan, chondroitin sulfate and deionized water. Obviously, the addition of a higher level of Tween 80 was effective in reducing the nanoemulsion size, however, a strong odor and possible health-related side effects may occur as well. Conversely, the large particle size of a nanoemulsion can be caused by the presence of components of large molecular weight. In addition, the zeta potential of the *C. osmophloeum* nanoemulsion was −42.6 mv, revealing the high stability of this nanoemulsion, as it was reported that the zeta potential has to be controlled and maintained at >30 mv or <−30 mv to enhance the nanoemulsion stability [45]. Moreover, the use of both lecithin and Tween 80 to prepare the nanoemulsion in our study may strengthen the repulsive force between particles. Additionally, lecithin was proven to be an optimal surfactant, as it showed high affinity towards cell membranes, and thus both the stability and bioavailability of the nanoparticles were elevated substantially [49].

The encapsulation efficiency of *C. osmophloeum* leaf nanoemulsion was determined to be 91.22%, which is higher than that reported by Tian et al. [50], Liu et al. [51] and Jo et al. [52], with the encapsulation efficiency being 81, 76.57 and 70%, respectively. The addition of PEG may play a vital role in increasing encapsulation efficiency of the *C. osmophloeum* leaf nanoemulsion, as it is capable of dissolving water-insoluble bioactive compounds such as cinnamaldehyde into oil for subsequent partition into the aqueous phase [53]. Additionally, PEG exhibits many merits, such as low toxicity, high water solubility and the formation of non-toxic metabolites through excretion from the body [54].

Table 7 shows the changes in particle size, PDI and zeta potential of *C. osmophloeum* leaf nanoemulsion during storage at 4 °C for 90 days and heating at 40, 70 and 100 °C for 2 h. Only a slight change in particle size, PDI and zeta potential was shown over a 90-day storage period at 4 °C and for heating at 100 °C for 2 h, implying that a high storage and heating stability of this nanoemulsion was successfully achieved. Though both particle size and zeta potential followed a time-dependent decline during heating, they are still within the acceptable range. The incorporation of PEG and lecithin into the formula may contribute to the greater stability of this nanoemulsion [49]. However, we have to point out that a turbid appearance occurred during heating, which may be caused by flocculation through nanoparticle interaction and aggregation, but after shaking, a transparent appearance occurred again. In addition, a high concentration of this nanoemulsion may also cause flocculation; this problem can be overcome by dilution. In a similar study, Zhang et al. [46] prepared clove/cinnamon essential oil nanoemulsion, and reported that a turbid appearance occurred with the heating temperature >90 °C, while a transparent appearance was shown at temperatures <90 °C. Apparently, temperature should also play a vital role in affecting the nanoemulsion stability.

### 3.7. Body Weight Gain, Food Intake and Water Intake

Table 8 shows the effects of various treatments on body weight gain, food intake and water intake of rats on a high-fat diet that received streptozotocin injection for diabetes induction. Following 1 week of feeding, the NC group was significantly higher (*p* < 0.05) than the DC group in terms of body weight. Moreover, after feeding for 4 weeks, the body weights of the NC group and the other treatments were significantly higher than the DC group. Compared to the DC group, the body weight was higher by 14.79, 12.75, 23.47, 26.02 and 31.12% for the HP, EL, EH, NL and NH groups, respectively. This result reveals that all the treatments, including cinnamon powder in hydrosol, cinnamon extract and nanoemulsion, were effective in reducing body weight loss in diabetic rats, with the high-dose nanoemulsion showing the most pronounced effect.

For food intake, only the EH and NH groups showed a decline, by 15.38 and 23.08%, respectively, compared to the DC group over a 4-week feeding period. Of the various treatments, the high-dose nanoemulsion was the most efficient in reducing food intake, and thus, the sense of starvation in diabetic rats was minimized. Similarly, after feeding for 4 weeks, the water intake for the HP, EL, NL, EH and NH groups was reduced by 31.03, 12.07, 46.55, 48.28 and 58.62%, respectively, when compared to the DC group. Comparatively, the high-dose nanoemulsion was the most prominent in reducing water intake, and thus, the feeling of thirst in diabetic rats was ameliorated.

In a previous study, Abdel-Halim et al. [54] prepared the extract and gold nanoextract (15 nm) from *Bauhinia variegata*, and reported that the body weight loss was reduced by 67.59 and 75.86%, respectively, in diabetic rats after feeding for 4 weeks. Likewise, Rani et al. [55] prepared thymoquinone extract and a nanoemulsion composed of gum rosin, lecithin, polyvinyl alcohol and polysorbate 80 with a mean particle size of 70.21 nm, and reported a reduction in body weight loss by 8.11–8.55% and 9.8–10.17%, respectively, in diabetic rats after 21 days of feeding. Furthermore, for the administration of diabetic rats with cinnamaldehyde at 20 mg/kg for 45 days, or water extract from cinnamon (3, 30, 100 mg/kg) for 22 days, the body weight loss was reduced by 15.15% [56] for the former and 4.67–22.33% for the latter [57]. By comparison, the nanoemulsion was more effective than the extract in reducing the body weight loss in diabetic rats.

### 3.8. Fasting Blood Glucose (FBG) and Oral Glucose Tolerance Test (OGTT)

Table 9 shows the effects of various treatments on the levels of FBG and OGTT in rats on a high-fat diet that received streptozotocin injection to induce diabetes. Following injection with streptozotocin for 1 week, the FBG levels were >200 mg/dL in rats for all the treatments except the NC group, implying a successful induction of diabetes in rats.

After the 4-week administration period, the DC group showed a significantly higher (*p* < 0.05) FBG level than the NC group, by 322.64%, while the FBG levels in diabetic rats were reduced by 21.39, 23.10, 34.00, 47.17 and 54.20%, respectively, for the HP, EL, EH, NL and NH groups when compared to the DC group. Similarly, following the 60-min administration period, the OGTT levels in diabetic rats were respectively diminished by 24.97, 31.62, 24.95, 47.42 and 55.73% for the HP, EL, EH, NL and NH groups when compared to the DC group, and further dropped to 30.81, 27.41, 45.77, 54.74 and 65.49% after administration for 120 min. By comparison, the high-dose nanoemulsion showed the most prominent effect in reducing both FBG and OGTT contents, followed by low-dose nanoemulsion and high-dose extract. Interestingly, the HP group was the least effective in reducing the FBG level, while the EL group was the least efficient in decreasing the OGTT level. This finding clearly demonstrates that the cinnamon nanoemulsion was more efficient than the cinnamon extract in reducing both FBG and OGTT contents, which may be due to the enhancement of β cell functions in the islets of Langerhans to secret insulin for the subsequent augmentation of insulin signal transduction to increase glucose intake in vivo. Additionally, cinnamaldehyde was shown to inhibit the activities of aldose reductase and α-glucosidase, and regulate the cellular response of insulin for the enhancement of glucose metabolism through the activation of insulin receptor kinase and glycogen synthase, as well as phosphorylation [58].

The anti-diabetic effects of cinnamon and its by-products have been well documented. For instance, following feeding of diabetic rats on a diet containing 5% cinnamon oil for 3 weeks, the FBG level was reduced by 48.71% [59]. Similarly, the FBG levels in diabetic rats declined respectively by 61.62 and 70.44% after administration of cinnamaldehyde at 20 mg/kg for 60 days [60] and 20 mg/kg for 45 days [50]. Additionally, a dose-dependent decline in FBG level was found after feeding diabetic rats diets containing different doses of cinnamon oil [61]. However, there is a lack of data regarding the reduction in FBG level as affected by the nanoemulsion prepared from cinnamon leaves and by-products. The following are examples of previous studies on the effects of nanoparticles/nanoemulsions on FBG reduction in diabetic rats. For example, the FBG levels in diabetic rats were shown to reduce by 54.56 and 59.81%, respectively, after feeding with Bauhinia extract and gold nanoextract for 4 weeks [54]. Likewise, the FBG contents were diminished by 39.46–50.49% and 46.75–58.27%, respectively, in diabetic rats following administration with thymoquinone extract and nanoemulsion for 21 days [55]. However, in a study investigating the effects of metformin and metformin-selenium nanoparticles (30–80 nm) on anti-diabetic activity in rats, the FBG levels were decreased only by 7.27 and 10.91%, respectively, after an 8-week feeding period [62]. This difference in anti-diabetic activity may be accounted for by the difference in feeding dose and period, as well as the components used to prepare nanoparticles/nanoemulsions and their size. It is worth pointing out that in many published reports dealing with the anti-diabetic activity of bioactive compounds using animal models, only the FBG level was determined, instead of OGTT.

Furthermore, nanoparticles were reported to block the p-glycoprotein efflux pump through the hardening of the lipid bilayer, while Fc-modified exenatide-loaded nanoparticles were shown to improve the hypoglycemic effect in mice through targeting the Fc-receptor of small intestine and colon cells [63]. Thus, the intake of the cinnamon nanoemulsion prepared in our study by pancreas cells may be enhanced to attain a possible passive targeting effect. The HOMA-IR often refers to the lower sensitivity of human liver, muscle and adipose cells toward insulin, making it difficult for blood glucose to enter cells for metabolism and energy supply; thus, more insulin must be secreted by the pancreas for hyperinsulinemia to occur. According to a report published by the University of Hong Kong in 2016 [64], HOMA-IR index values between 1.4 and 2.0 can be used to distinguish abnormal blood sugar and type 2 diabetes, for which high risk is indicated by HOMA-IR index values >2.

Table 9 also shows the changes in the serum insulin and HOMA-IR index of rats as affected by various treatments. A significantly higher (*p* < 0.05) insulin level was shown for the DC group compared to the NC group. Moreover, for the HP, EL, NL, EH and NH groups, the serum insulin levels in rats declined by 8.89, 14.44, 25.56, 32.22 and 47.78%, respectively, compared to the DC group. Comparatively, the high-dose nanoemulsion was the most effective in reducing the serum insulin level in rats, followed by high-dose extract, low-dose nanoemulsion, low-dose extract and cinnamon powder in hydrosol. Similarly, compared to the DC group, the HOMA-IR index for HP, EL, NL, EH and NH groups decreased by 23.60, 38.16, 31.23, 62.67 and 75.95%, respectively, revealing that both high-dose nanoemulsion and high-dose extract were the most efficient in diminishing the HOMA-IR index.

In another study involving a clinical trial, Anderson et al. [65] reported that for people with elevated serum glucose, the intake of cinnamon water extract at 500 mg/day for 2 months was effective in lowering serum insulin and HOMA-IR by 7.83 and 13.96%, respectively. Likewise, for women with polycystic ovary syndrome, the HOMA-IR declined by 44.5% after cinnamon consumption for 8 weeks [66], while for patients with type 2 diabetes mellitus, the serum insulin and HOMA-IR was lowered by 1.77 mIU/L and 1.01, respectively, following the intake of cinnamon at 1g/day for 3 months [67]. However, for the animal experiment, the serum insulin was respectively reduced by 11.45 and 12.8% after feeding diabetic rats with metformin and metformin–nanoselenium for 8 weeks, and the HOMA-IR dropped by 17.92 and 22.41%. It was postulated that the administration of selenium nanoparticles may induce a rise in selenium protein and result in the elevation of the sensitivity of the insulin signal transduction route. In addition, the presence of phenolic compounds in cinnamon extract was found to increase phosphoinositide 3-kinase activity for the subsequent elevation of glucose transporter 4 expression and the attenuation of inhibiting glycogen synthase, leading to the promotion of glucose transportation within cells and increase in glycogen synthesis so that glucose utilization was raised [68]. Nevertheless, the increase in bioavailability through administration of nanoparticle/nanoemulsion may also play a vital role in enhancing the treatment efficiency of diabetes mellitus.

### 3.9. Serum Biochemical Parameters

The serum biochemical parameters in rats, including TC, TG, AST, ALT, UA, BUN and CREA, are shown in Table 10. Compared to the DC group, the NC group showed a significantly lower (*p* < 0.05) level of TC, while for the HP, EL, NL, EH and NH groups, the TC levels were reduced by 10.75, 19.59, 19.24, 29.29 and 32.93%, respectively, implying that both high-dose extract and high-dose nanoemulsion were the most efficient in reducing the serum TC level. Similar results were observed for TG, as evident by reductions of 23.72, 26.80, 30.35, 41.18 and 48.74% for the HP, EL, NL, EH and NH groups, respectively, with both high-dose extract and high-dose nanoemulsion showing the most prominent effects in lowering the serum TG level. Thus, the intake of high-dose extract or nanoemulsion may be effective in improving cardiovascular function. Similar findings were observed by Babu et al. [56] and Al-Logmani [59], as a significant reduction in both TC and TG in diabetic rats was shown after the administration of cinnamon oil or cinnamon extract.

The effects of various treatments on the serum AST and ALT levels in diabetic rats are also shown in Table 10. The NC group showed a significantly lower (*p* < 0.05) AST level than the DC group, while for the HP, EL, NL, EH and NH groups, the AST levels were decreased by 33.66, 45.87, 41.73, 46.07 and 47.64%, respectively. A similar outcome was found for ALT, with the levels being declined by 22.91, 30.00, 31.25, 56.04 and 46.88%, respectively, for the HP, EL, NL, EH and NH groups. Collectively, the intake of high-dose extract or nanoemulsion may be effective in improving liver function. Similar results were reported by Abdel-Halim et al. [54], Babu et al. [56] and Al-Logmani [59], as a significant rise in both AST and ALT levels was shown in diabetic rats following feeding with cinnamon oil or extract.

Table 10 also shows the effects of various treatments on the levels of UA, BUN and CREA in diabetic rats. Compared to the DC group, the NC group showed a significantly lower (*p* < 0.05) UA level, while for the HP, EL, NL, EH and NH groups, the UA levels declined insignificantly (*p* > 0.05) by 8.23, 10.49, 5.56, 1.03 and 13.79%, respectively. However, for BUN, the levels were significantly decreased (*p* < 0.05) by 30.41, 35.04, 39.68, 48.96 and 51.54%, respectively, for the HP, EL, NL, EH and NH groups when compared to the DC group. Similar to UA, compared to the DC group, the CREA levels were insignificantly decreased (*p*>0.05) by 45.40, 10.92, 28.16, 37.93 and 39.66%, respectively, for the HP, EL, NL, EH and NH groups. Taken together, the consumption of high-dose extract or nanoemulsion may be efficient in improving kidney function. In several previous studies, significant reductions in UA or BUN levels in diabetic rats were shown following the administration of cinnamon oil [59,61]. However, both BUN and CREA levels were found to rise while the UA level declined in diabetic rats after intake of cinnamaldehyde at 20 mg/kg bw for 60 days [60]. Apparently the increase or decrease in BUN and CREA levels may be associated with the type of material, dose, feeding period and physiological characteristics of rats. Singh et al. [69] further pointed out that a high FBG level can lead to proteinuria through the elevation of the angiotensin II concentration in cells and overexpression of the transforming growth factor-β (TGF-β).

All in all, both cinnamon extract and nanoemulsion followed a dose-dependent response in improving lipid, liver and kidney functions in diabetic rats. By comparison, the high-dose nanoemulsion was the most effective, probably because of the encapsulation of bioactive compounds such as cinnamaldehyde in the nanoemulsion for prolonged circulation and maintenance of effective concentrations in vivo. Moreover, compared to traditional oral intake, encapsulated insulin nanoparticles were previously reported to resist gastric degradation, while encapsulated insulin was released automatically in vivo for the treatment of hyperglycemia [70].

In most human diseases, oxidative stress plays a key role in pathogenesis, which occurs due to an imbalance between reactive oxygen species (ROS) and antioxidants [10]. Diabetes mellitus is a metabolic disorder caused by insufficient insulin secretion and resistance, resulting in hypoglycemia or hyperglycemia. Such poorly controlled blood glucose is closely associated with elevated levels of ROS, resulting in damage to the cell membrane through their reaction with proteins, lipids, DNA and biomolecules [11]. Moreover, ROS production stimulates the generation of proinflammatory cytokines such as interleukin-6 (IL-6), interleukin-1β (IL-1β), tumor necrosis factor-alpha (TNF-α) and nitric oxide (NO) in diabetic patients [12]. Obviously, patients with type 2 diabetes are prone to the elevation of oxidative stress, resulting in reduced antioxidant activity. Hsu et al. [11] investigated the in vivo antioxidant activity of the leaves of *C. osmophloeum*, and reported that the major constituents, cinnamaldehyde and camphor, could induce antioxidative gene expressions, such as SOD and GST, to lower juglone-induced oxidative stress in nematodes (*Caenorhabditis elegans*). In a later study, Lee et al. [12] evaluated the hypoglycemic and pancreas-protective effects of essential oil from the leaves of *C. osmophloeum* in streptozotocin-induced diabetic rats, reporting that *C. osmophloeum* leaves could lower fasting blood glucose and fructosamine levels, accompanied by elevating plasma and pancreatic insulin levels under a fasting condition, through a reduction in the levels of thiobarbituric acid reactive substances in the pancreas, IL-1β and NO, as well as a rise in the activities of SOD and glutathione reductase. Such lowered oxidative stress and proinflammatory cytokines implied a protective effect on pancreatic β cells by antioxidant compounds from the leaves of *C. osmophloeum*. Moreover, Sahib et al. [10] have shown that an intake of 1 g of cinnamon for 12 weeks reduced fasting blood glucose and glycosylated hemoglobin among poorly controlled type 2 diabetic patients through the enhancement of antioxidant activities, such as SOD and glutathione in serum. The major cinnamaldehyde compound was also demonstrated to alleviate the levels of leptin, TNF-α, malondialdehyde and NO, with a concomitant increase in GSH and CAT activities in streptozotocin-induced gestational diabetic rats [13]. The foregoing discussion revealed that the antioxidants in cinnamon, including cinnamaldehyde, phenolic acids and flavonoids, could reduce the pathological damage of pancreas β cells via attenuation of oxidative stress and proinflammatory response for effective treatment of diabetes.

In this study, a total of 15 bioactive compounds were determined in the leaves of Taiwan *C. osmophloeum* by UPLC-MS/MS, with cinnamaldehyde being predominant. Upon encapsulating the leaf extract of *C. osmophloeum* into a stable nanoemulsion system, the high-dose nanoemulsion was demonstrated to be the most effective in reducing fasting blood glucose and oral glucose tolerance test values as well as lowering serum insulin and the homeostatic model assessment of insulin resistance index in streptozotocin-induced diabetic rats.

The name cinnamon by itself suggests that it is rich in bioactive compounds such as cinnamaldehyde, cinnamic acid and cinnamoyl alcohol, all of which are shown to be present in high quantities in the Taiwan-based cinnamon variety *C. osmophloeum* in this study (Table 4). Although cinnamaldehyde has been used traditionally as a natural flavoring and fragrance agent, the accumulating research reports over the years reveal that it exhibits beneficial effects in the prevention and treatment of diabetes-related abnormalities by regulating blood glucose and lipid metabolism as well as through the enhancement of insulin sensitivity [71,72]. More specifically, cinnamaldehyde has been shown to improve several diabetic disorders associated with different organs and tissues including liver, kidney, pancreas, gastrointestinal tract, skeletal muscle tissue, hypothalamus and adipose tissue [71]. It lowers glycolipid levels in diabetic animals by increasing glucose uptake and improving insulin sensitivity in adipose and skeletal muscle tissues, enhancing glycogen synthesis in the liver, restoring pancreatic islets dysfunction, delaying gastric emptying rates and alleviating diabetic renal and brain disorders [71]. The underlying mechanism for the exertion of these functions may involve the regulation of multiple signaling pathways, including PPARs, AMPK, P13K/IRS-1, RBP4-GLUT4, ERK/JNK/p38MAPK, TRPA1-ghrelin and Nrf2 [71,72]. In addition, as discussed above, cinnamaldehyde and the other bioactive compounds determined in this study can ameliorate oxidative stress by preventing the glycation of antioxidant enzymes such as SOD, CAT and glutathione peroxidase, facilitating pancreatic islet regeneration through the protection of β cells from free radical insults and enabling insulin secretion [8,9,10,11,12,13]. Furthermore, based on their structural characteristics, flavonoids and phenolic acids can stabilize free radicals through hydrogen donation by hydroxy groups and chelation of metal ions (prooxidants) by both hydroxy and carboxy groups [73]. Thus, the extremely high concentration of cinnamaldehyde in the leaves of Taiwan *C. osmophloeum* can significantly reduce its effective dose for providing high antioxidant activity and eventually attaining an enhanced therapeutic effect.

## 4. Conclusions

In conclusion, an UPLC-MS/MS method was employed to analyze 15 bioactive compounds in cinnamon leaves with high accuracy and precision. The nanoemulsion, composed of soybean oil, Tween 80, lecithin, PEG and deionized water, was successfully prepared with the mean particle size of 36.58 nm, zeta potential of −42.6 mV and polydispersity index of 0.222. A high stability for this nanoemulsion when stored at 4 °C for 3 months and heated at 100 °C for 2 h was discovered. The animal experiment revealed that the levels of FBG, insulin and HOMA-IR were reduced by 21.39–54.20%, 8.89–47.78% and 23.60–75.95%, respectively, in diabetic rats for all the hydrosol, extract and nanoemulsion treatments, while the OGTT was improved substantially. In addition, the levels of TC, TG, AST, ALT, UA, BUN and CREA in diabetic rats were declined. Taken together, the high-dose cinnamon nanoemulsion was the most effective in improving the functions of diabetic rats, and possesses great potential to be developed into a health food or botanic drug in the future.

## Figures and Tables

**Table 1:** The composition of fresh and dried *C. osmophloeum* leaves ^a^.

	Moisture (%)	Ash (%)	Crude Protein (%)	Crude Fat (%)	Carbohydrate (%)
Fresh	47.73 ± 0.50	2.23 ± 0.21	6.20 ± 0.10	1.17 ± 0.29	42.67 ± 0.31
Dried	5.33 ± 0.12	5.03 ± 0.29	14.80 ± 0.80	2.17± 0.24	72.67 ± 0.54

^a^ Values are mean ± standard deviation of triplicate determinations.

**Table 2:** Effect of pure water and different ethanol proportion on the contents of total phenolic acids and total flavonoids in *C. osmophloeum* leaves ^A^.

	100% H_2_O	30% EtOH	50% EtOH	70% EtOH
Total phenolic acids ^B^	19.08 ± 2.07 ^b^	23.54 ± 1.66 ^a^	20.96 ± 1.78 ^ab^	19.6 ± 10.24 ^b^
Total flavonoids ^C^	13.98 ± 0.88 ^c^	27.90 ± 0.85 ^a^	24.73 ± 0.77 ^b^	11.67 ± 0.33 ^d^

^A^ Data are presented as mean ± standard deviation of triplicate determinations, and data with different lower case letters (a–d) in the same row are significantly different at *p* < 0.05; ^B^ Data are expressed as mg/g of gallic acid equivalent (GAE); ^C^ Data are expressed as mg/g of quercetin equivalent (QE).

**Table 3:** Effect of different drying methods on the contents of total phenolic acids and total flavonoids in *C. osmophloeum* leaves ^A^.

	Fresh	Freeze Drying ^D^	Oven Drying ^E^	Baking ^F^
Total phenolic acids ^B^	9.27 ± 1.02 ^b^	22.03 ± 1.40 ^a^	23.54 ± 1.66 ^a^	21.82 ± 1.17 ^a^
Total flavonoids ^C^	9.81 ± 1.09 ^b^	27.84 ± 0.45 ^a^	27.90 ± 0.85 ^a^	26.04 ± 2.12 ^a^

^A^ Data are presented as mean ± standard deviation of triplicate determinations, and data with different lower case letters (a,b) in the same row are significantly different at *p* < 0.05; ^B^ Data are expressed as mg/g of gallic acid equivalent (GAE); ^C^ Data are expressed as mg/g quercetin equivalent (QE); ^D^ Dried at −45 °C and vacuum degree of 125 mT for 4 days; ^E^ Dried at 60 °C for 2 h; ^F^ Baked at 60 °C for 3 h.

**Table 4:** Identification and quantitation data of cinnamaldehyde and the other bioactive compounds in *C. osmophloeum* leaves and hydrosols by UPLC-MS/MS.

Peak no.	Compound ^a^	Retention Time (min)	MS/MS (m/z)			Content		
			Precursor Ion	Product Ion	Reported	Leaves (µg/g) ^a,f^	Leaves (µg/g) ^a,g^	Hydrosols (mg/L) ^h^
1	Quercetin	7.35	301	151	301,151 ^b^	1.85 ± 0.14	4.81 ± 0.10	ND ^i^
2	Coumarin	5.60	147	91	147, 91 ^d^	2.46 ± 0.04	1.11 ± 0.03	ND
3	Quercetin-3-*O*-galactoside	7.34	463	300	463, 300 ^b^	2.64 ± 0.20	4.11 ± 0.08	ND
4	Quercetin-3-*O*-glucoside	7.34	463	300	463, 301 ^b^	2.64 ± 0.20	4.11 ± 0.08	ND
5	Rutin	7.29	609	300	609, 300 ^b^	1.71 ± 0.01	1.45 ± 0.05	ND
6	Caffeic acid	4.25	179	134	179, 135 ^b^	2.52 ± 0.10	9.37 ± 0.13	ND
7	Benzoic acid	6.57	121	77	121, 77 ^b^	43.06 ± 0.04	22.98 ± 0.20	0.39 ± 0.01
8	5-*O*-Caffeoylquinic acid	2.18	353	179	353, 179 ^b^	0.56 ± 0.04	0.34 ± 0.02	ND
9	Cinnamyl alcohol	8.17	117	115	117, 115 ^c^	91.88 ± 2.03	139.60 ± 2.41	3.55 ± 1.02
10	*p*-Coumaric acid	5.60	164	90	163, 91 ^e^	1.03 ± 0.04	0.87 ± 0.07	ND
11	Kaempferol 3-β-D-glucopyranoside	8.40	447	284	447, 285 ^b^	21.09 ± 0.97	14.62 ± 0.64	ND
12	Eugenol	10.70	165	137	165, 137 ^b^	49.27 ± 1.28	5.26 ± 0.15	12.06 ± 0.15
13	Kaempferol	8.57	287	153	287, 153 ^b^	15.12 ± 0.41	5.55 ± 0.24	ND
14	Cinnamaldehyde	8.37	132	55	132, 55 ^c^	5240.00 ± 29.44	295.11 ± 8.43	1435.00 ± 68.69
15	*trans*-Cinnamic acid	9.08	147	103	146, 103 ^c^	292.80 ± 12.18	253.8 ± 9.72	0.90 ± 0.06

^a^ All the compounds (1–15) were positively identified based on the comparison of retention times and mass spectra with those of reference standards; ^b^ Reported MS/MS data are derived from Vallverdú-Queralt et al. [36]; ^c^ Reported MS/MS data are derived from Ji et al. [37]; ^d^ Reported MS/MS data are derived from Arigò et al. [38]; ^e^ Reported MS/MS data are derived from Flores et al. [39]; ^f^ With 80% ethanol as the extraction solvent; ^g^ With 30% ethanol as the extraction solvent; ^h^ Directly analyzed without solvent extraction; ^i^ ND, not detected.

**Table 5:** Quality control data of cinnamaldehyde and the other bioactive compounds in *C. osmophloeum* leaves by UPLC-MS/MS.

Compound	LOD (ng/g) ^a^	LOQ(ng/g) ^b^	Intra-Day Variability ^c^		Inter-Day Variability ^c^	
			Mean ± SD (µg/g)	RSD (%) ^d^	Mean ± SD (µg/g)	RSD (%) ^d^
Quercetin	8.40	25.19	1.89 ± 0.13	7.41	1.89 ± 0.10	5.86
Coumarin	0.85	2.56	2.36 ± 0.14	6.37	2.23 ± 0.16	7.79
Quercetin-3-*O*-galactoside	1.74	5.21	2.71 ± 0.17	6.83	2.21 ± 0.19	8.93
Quercetin-3-*O*-glucoside	1.74	5.21	2.71 ± 0.17	6.83	2.21 ± 0.19	8.93
Rutin	0.90	2.69	1.70 ± 0.04	2.69	1.68 ± 0.07	4.44
Caffeic acid	1.41	4.23	2.63 ± 0.14	5.55	2.61 ± 0.13	5.44
Benzoic acid	1.50	4.50	42.24 ± 0.93	2.33	42.4 ± 1.26	3.16
5-*O*-Caffeoylquinic acid	1.33	4.00	0.55 ± 0.04	7.66	0.55 ± 0.04	7.48
Cinnamyl alcohol	8.08	24.24	92.58 ± 1.69	1.93	92.45 ± 1.58	1.81
*p*-Coumaric acid	2.13	6.39	0.98 ± 0.08	8.21	0.99 ± 0.05	5.87
Kaempferol 3-β-D- glucopyranoside	0.64	1.93	21.07 ± 1.22	6.14	21.29 ± 1.26	6.27
Eugenol	6.23	8.68	50.28 ± 2.00	4.21	50.49 ± 1.45	3.04
Kaempferol	0.14	0.43	15.35 ± 0.82	5.70	15.35 ± 0.82	5.70
Cinnamaldehyde	0.08	0.24	5135.78 ± 139.88	2.89	5141.33 ± 142.92	2.95
*trans*-Cinnamic acid	0.19	0.56	291.16 ± 10.28	3.74	287.04 ± 11.81	4.36

^a^ Limit of detection based on S/N ≥ 3; ^b^ Limit of quantitation based on S/N ≥ 10; ^c^ Mean of triplicate analyses ± standard deviation; ^d^ Relative standard deviation (%) = (standard deviation/mean) × 100.

**Table 6:** Recovery of cinnamaldehyde and the other bioactive compounds in *C. osmophloeum* leaves by UPLC-MS/MS.

Compound	Original (µg) ^a^	Spiked (µg) ^b^	Found (µg) ^c^	Recovery (%) ^d^	Mean ± SD (%)	RSD (%) ^e^
Quercetin	0.394	0.1	0.492	99.60	96.78 ± 3.97	4.11
		1	1.310	93.97		
Coumarin	0.486	0.1	0.587	100.17	100.15 ± 0.03	0.03
		1	1.488	100.13		
Quercetin-3-*O*-galactoside	0.500	0.1	0.592	98.67	98.57 ± 0.14	0.14
		1	1.477	98.47		
Quercetin-3-*O*-glucoside	0.500	0.1	0.592	98.67	98.57 ± 0.14	0.14
		1	1.477	98.47		
Rutin	0.336	0.1	0.441	101.15	104.88 ± 5.28	5.03
		1	1.451	108.61		
Caffeic acid	0.484	1	1.475	99.39	104.22 ± 6.83	6.55
		10	11.433	109.05		
Benzoic acid	8.494	1	9.564	100.74	100.76 ± 0.03	0.03
		10	18.638	100.78		
5-O-Caffeoylquinic acid	0.120	0.1	0.218	99.09	102.76 ± 5.19	5.05
		1	1.192	106.43		
Cinnamyl alcohol	18.186	10	29.052	103.07	107.30 ± 5.98	5.57
		100	131.811	111.53		
p-Coumaric acid	0.192	0.1	0.292	100.00	102.56 ± 3.62	3.53
		1	1.253	105.12		
Kaempferol 3-β-D-glucopyranoside	4.494	1	5.473	99.62	106.62 ± 9.90	9.28
		10	16.467	113.61		
Eugenol	10.366	10	20.818	102.22	96.67 ± 7.85	8.12
		100	100.567	91.12		
Kaempferol	3.134	1	4.049	97.94	105.10 ± 10.12	9.63
		10	14.744	112.26		
Cinnamaldehyde	1020	1000	1907.333	94.42	90.21 ± 5.95	6.60
		10,000	9477.333	86.00		
trans-Cinnamic acid	56.734	10	67.177	100.66	101.11 ± 0.63	0.63
		100	159.18	101.56		

^a^ Quantity of each bioactive compound present originally in 0.2 g of *C. osmophloeum* leaf powder; ^b^ Quantity of each standard spiked into 0.2 g of *C. osmophloeum* leaf powder; ^c^ Quantity of each bioactive compound extracted and analyzed by UPLC-MS/MS from 0.2 g of *C. osmophloeum* leaf powder containing original content plus spiked standard; ^d^ Recovery (%) = ((amount found—original amount)/amount spiked) x 100; ^e^ Relative standard deviation (%) = (standard deviation/mean) × 100.

**Table 7:** The average particle size, polydispersity index and zeta potential changes in *C. osmophloeum* leaf nanoemulsion during storage for 90 days at 4 °C and during heating at 40 °C, 70 °C and 100 °C for varied time length.

Temp	Particle Size (nm)					Polydispersity Index						Zeta Potential (mV)			
	0 h	0.5 h	1 h	1.5 h	2 h	0 h	0.5 h	1 h	1.5 h	2 h	0 h	0.5 h	1 h	1.5 h	2 h
40 °C	36.58	36.92	36.23	36.46	36.68	0.222	0.262	0.266	0.276	0.279	−42.6	−40.50	−39.50	−39.23	−38.47
70 °C	36.58	35.23	35.73	35.31	33.34	0.222	0.285	0.249	0.268	0.263	−42.6	−38.90	−37.37	−36.57	−36.47
100 °C	36.58	33.34	33.60	31.03	30.31	0.222	0.234	0.304	0.320	0.324	−42.6	−37.57	−36.37	−36.57	−35.8

^a^ Data are shown as mean of triplicate analyses; ^b^ Data are shown as mean ± standard deviation of triplicate analyses.

**Table 8:** Effects of *C. osmophloeum* leaf extract, nanoemulsion and hydrosol on body weight, food intake and water intake of rats on a high-fat diet that received streptozotocin injection for diabetes induction.

Group	Body Weight (g/rat)		Food Intake (g/day/rat)		Water Intake (g/day/rat)	
	0 Week	4 Weeks	0 Week	4 Weeks	0 Week	4 Weeks
NC	215.00 ± 9.57 ^Ab^	406.67 ± 11.06 ^Aa^	17.50 ± 5.00 ^Ab^	32.50 ± 11.18 ^Aa^	35.00 ± 12.25 ^Ab^	46.25 ± 10.90 ^BCa^
DC	211.67 ± 6.87 ^Ab^	326.67 ± 20.55 ^Ca^	18.75 ± 2.50 ^Ab^	32.50 ± 8.66 ^Aa^	31.25 ± 4.33 ^Ab^	72.50 ± 50.25 ^Aa^
HP	213.33 ± 9.43 ^Ab^	375.00 ± 23.63 ^Ba^	16.25 ± 5.59 ^Ab^	32.50 ± 11.18 ^Aa^	35.00 ± 12.25 ^Ab^	50.00 ± 7.07 ^ABCa^
EL	212.50 ± 8.04 ^Ab^	368.33 ± 21.15 ^Ba^	15.63 ± 7.40 ^Ab^	33.75 ± 4.33 ^Aa^	37.50 ± 15.00 ^Ab^	63.75 ± 38.97 ^ABa^
NL	220.00 ± 10.00 ^Ab^	403.33 ± 20.55 ^Aa^	15.00 ± 6.12 ^Ab^	32.50 ± 5.00 ^Aa^	33.75 ± 19.20 ^Ab^	38.75 ± 22.78 ^Ca^
EH	220.00 ± 5.77 ^Ab^	411.67 ± 22.67 ^Aa^	17.50 ± 9.35 ^Ab^	27.50 ± 5.00 ^ABa^	35.00 ± 7.07 ^Aa^	37.50 ± 16.58 ^Ca^
NH	211.67 ± 12.13 ^Ab^	428.33 ± 26.09 ^Aa^	14.38 ± 5.45 ^Ab^	25.00 ± 7.07 ^Ba^	31.25 ± 17.85 ^Aa^	30.00 ± 12.25 ^Ca^

Data are presented as mean ± standard deviation (n = 8). Values with different capital letters (A–C) in the same column and lower case letters (a,b) in the same row are significantly different at *p* < 0.05. NC, normal control group; DC, high-fat diet with streptozotocin injection at a dose of 65 mg/kg bw; HP, high-fat diet with streptozotocin injection at a dose of 65 mg/kg bw and administration of hydrosols at a dose of 10 mL/kg bw and leaf powder at a dose of 0.5 g/kg bw; EL, high-fat diet with streptozotocin injection at a dose of 65 mg/kg bw and administration of leaf extract at a dose of cinnamaldehyde 20 mg/kg bw; NL, high-fat diet with streptozotocin injection at a dose of 65 mg/kg bw and administration of nanoemulsion at a dose of cinnamaldehyde 20 mg/kg bw; EH, high-fat diet with streptozotocin injection at a dose of 65 mg/kg bw and administration of leaf extract at a dose of cinnamaldehyde 60 mg/kg bw; NH, high-fat diet with streptozotocin injection at a dose of 65 mg/kg bw and administration of nanoemulsion at a dose of cinnamaldehyde 60 mg/kg bw.

**Table 9:** Effects of administration of *C. osmophloeum* leaf extract, nanoemulsion and powder in hydrosol on fasting blood glucose (FBG), oral glucose tolerance test (OGTT), serum insulin and HOMA-IR index in high-fat diet with streptozotocin injection into diabetic rats.

Group	FBG (mg/dL)		OGTT (mg/dL)				Insulin (μg/L)	HOMA-IR
	0 Week	4 Weeks	0 min	30 min	60 min	120 min	4 Weeks	4 Weeks
NC	103.33 ± 9.07 ^Aa^	106.17 ± 7.99 ^Ea^	107.00 ± 7.16 ^Ea^	109.00 ± 7.16 ^Ea^	104.67 ± 7.63 ^Ea^	98.00 ± 5.80 ^Ea^	0.44 ± 0.05 ^C^	2.91 ± 0.31 ^E^
DC	105.50 ± 8.79 ^Ab^	448.00 ± 47.15 ^Aa^	434.33 ± 92.26 ^Ab^	590.50 ± 17.53 ^Aa^	591.83 ± 18.26 ^Aa^	584.83 ± 19.92 ^Aa^	0.90 ± 0.17 ^A^	24.24 ± 8.32 ^A^
HP	103.00 ± 5.35 ^Ab^	352.17 ± 59.54 ^Ba^	359.50 ± 108.80 ^ABc^	394.50 ± 153.62 ^Bb^	444.00 ± 156.16 ^Ba^	404.67 ± 122.86 ^Bb^	0.82 ± 0.13 ^AB^	18.52 ± 7.85 ^AB^
EL	96.67 ± 5.12 ^Ab^	344.5 ± 44.94 ^BCa^	320.33 ± 68.40 ^BCb^	399.50 ± 65.52 ^Bab^	444.17 ± 55.06 ^Ba^	424.50 ± 47.39 ^Ba^	0.77 ± 0.29 ^AB^	14.99 ± 5.52 ^BC^
NL	101.50 ± 9.38 ^Ab^	295.67 ± 46.79 ^Ca^	283.17 ± 66.00 ^BCDc^	326.83 ± 53.31 ^Cbc^	392.17 ± 126.35 ^Ba^	317.17 ± 52.65 ^Cbc^	0.67 ± 0.24 ^ABC^	16.67 ± 6.11 ^CD^
EH	104.50 ± 8.52 ^Ab^	236.67 ± 33.67 ^Da^	244.33 ± 46.84 ^CDb^	300.50 ± 68.74 ^Ca^	311.17 ± 38.88 ^Ca^	264.67 ± 46.72 ^CDb^	0.61 ± 0.34 ^BC^	9.05 ± 4.86 ^CDE^
NH	101.17 ± 2.27 ^Ab^	205.17 ± 22.57 ^Da^	202.83 ± 22.67 ^Db^	241.83 ± 38.55 ^Da^	256.50 ± 42.96 ^Da^	201.83 ± 26.67 ^Db^	0.47 ± 0.06 ^C^	5.83 ± 0.94 ^DE^

Data are presented as mean ± standard deviation (n = 8). Values with different capital letters (A–E) in the same column and small letters (a–c) in the same row for FBG or OGTT are significantly different at *p* < 0.05. HOMA-IR, homeostatic model assessment of insulin resistance; NC, normal control group; DC, high-fat diet with streptozotocin injection at a dose of 65 mg/kg bw; HP, high-fat diet with streptozotocin injection at a dose of 65 mg/kg bw and administration of powder in hydrosols at a dose of 10 mL/kg bw; EL, high-fat diet with streptozotocin injection at a dose of 65 mg/kg bw and administration of leaf extract at a dose of cinnamaldehyde 20 mg/kg bw; NL, high-fat diet with streptozotocin injection at a dose of 65 mg/kg bw and administration of nanoemulsion at a dose of cinnamaldehyde 20 mg/kg bw; EH, high-fat diet with streptozotocin injection at a dose of 65 mg/kg bw and administration of leaf extract at a dose of cinnamaldehyde 60 mg/kg bw; NH, high-fat diet with streptozotocin injection at a dose of 65 mg/kg bw and administration of nanoemulsion at a dose of cinnamaldehyde 60 mg/kg bw.

**Table 10:** Effects of the administration of *C. osmophloeum* leaf extract, nanoemulsion and hydrosol for 4 weeks on serum biochemical parameters of rats on a high-fat diet that received streptozotocin injection to induce diabetes.

Group	Serum Biochemical Parameters						
	TC (mg/dL)	TG (mg/dL)	AST (U/L)	ALT (U/L)	UA (mg/dL)	BUN (mg/dL)	CREA (mg/dL)
NC	74.83 ± 13.11 ^B^	96.17 ± 19.96 ^CD^	36.17 ± 10.93 ^B^	44.17 ± 4.71 ^CD^	6.13 ± 1.50 ^B^	17.67 ± 0.75 ^BCD^	0.30 ± 0.10 ^B^
DC	96.17 ± 18.04 ^A^	178.50 ± 37.93 ^A^	84.67 ± 29.5 ^A^	80.00 ± 21.94 ^A^	8.10 ± 2.09 ^A^	32.33 ± 4.82 ^A^	0.58 ± 0.28 ^A^
HP	85.83 ± 16.34 ^AB^	136.17 ± 20.46 ^B^	56.17 ± 26.13 ^AB^	61.67 ± 17.99 ^B^	7.43 ± 1.86 ^AB^	22.50 ± 4.79 ^B^	0.32 ± 0.24 ^AB^
EL	77.33 ± 19.69 ^ABC^	130.67 ± 18.71 ^B^	45.83 ± 29.66 ^B^	56.00 ± 13.4 ^BC^	7.25 ± 0.81 ^AB^	21.00 ± 3.46 ^BC^	0.52 ± 0.23 ^AB^
NL	77.67 ± 14.13 ^ABC^	124.33 ± 32.78 ^BC^	49.33 ± 13.01 ^B^	55.00 ± 10.13 ^BC^	7.65 ± 1.11 ^AB^	19.50 ± 7.30 ^BCD^	0.42 ± 0.12 ^AB^
EH	68.00 ± 8.27 ^BC^	105.0 ± 17.88 ^BCD^	45.67 ± 24.82 ^B^	35.17 ± 7.71 ^D^	8.02 ± 0.56 ^A^	16.50 ± 1.80 ^CD^	0.36 ± 0.10 ^AB^
NH	64.50 ± 13.21 ^C^	91.50 ± 12.84 ^D^	44.33 ± 20.37 ^B^	42.50 ± 10.08 ^CD^	6.98 ± 1.40 ^AB^	15.67 ± 2.36 ^D^	0.35 ± 0.13 ^AB^

Data are presented as mean ± standard deviation (n = 8). Values with different capital letters (A–D) in the same column are significantly different at *p* < 0.05. NC, normal control group; DC, high-fat diet with streptozotocin injection at a dose of 65 mg/kg bw; HP, high-fat diet with streptozotocin injection at a dose of 65 mg/kg bw and administration of hydrosols at a dose of 10 mL/kg bw and leaf powder at a dose of 0.5 g/kg bw; EL, high-fat diet with streptozotocin injection at a dose of 65 mg/kg bw and administration of leaf extract at a dose of cinnamaldehyde 20 mg/kg bw; NL, high-fat diet with streptozotocin injection at a dose of 65 mg/kg bw and administration of nanoemulsion at a dose of cinnamaldehyde 20 mg/kg bw; EH, high-fat diet with streptozotocin injection at a dose of 65 mg/kg bw and administration of leaf extract at a dose of cinnamaldehyde 60 mg/kg bw; NH, high-fat diet with streptozotocin injection at a dose of 65 mg/kg bw and administration of nanoemulsion at a dose of cinnamaldehyde 60 mg/kg bw; TC, total cholesterol; TG, triglyceride; AST, aspartate aminotransferase; ALT, alanine aminotransferase; UA, uric acid; BUN, blood urea nitrogen; CREA, creatinine.

## Data Availability

Data is contained within the article or Supplementary Materials.

## Supplementary Materials

The following supporting information can be downloaded at: https://www.mdpi.com/2076-3921/12/1/29/s1, Materials section with the details of reagents and instruments used in this study; Table S1: The linear regression equation and coefficient of determination (R^2^) obtained from the calibration curves prepared for 15 standard bioactive compounds.
