# Correction: Huang, Y.-C.; Chen, B.-H. A Comparative Study on Improving Streptozotocin-Induced Type 2 Diabetes in Rats by Hydrosol, Extract and Nanoemulsion Prepared from Cinnamon Leaves. *Antioxidants* 2023, *12*, 29

**DOI:** 10.3390/antiox12101799

**Published:** 2023-09-25

**Authors:** Yu-Chi Huang, Bing-Huei Chen

**Affiliations:** 1 Department of Food Science, Fu Jen Catholic University, New Taipei City 24205, Taiwan; jyjariel0324@gmail.com; 2 Department of Nutrition, China Medical University, Taichung 40402, Taiwan

## Error in Table

In the original publication [1], there was a mistake in the Table 9 caption as published: ***‘Effects of administration of C. osmophloeum leaf extract, nanoemulsion and hydrosol on fasting blood glucose (FBG), oral glucose tolerance test (OGTT), serum insulin and HOMA-IR index in rats on a high-fat diet that received streptozotocin injection to induce diabetes (Table 9)’***. More specifically, **there was a printing mistake in Table 9, with column 4 being duplicated in columns 5 through 7**. The corrected caption appears below: ‘Effects of administration of *C. osmophloeum* leaf extract, nanoemulsion and powder in hydrosol on fasting blood glucose (FBG), oral glucose tolerance test (OGTT) serum insulin and HOMA-IR index in high-fat diet with streptozotocin injection into diabetic rats’. The authors state that the scientific conclusions are unaffected. This correction was approved by the Academic Editor. The original publication has also been updated.

## Figures and Tables

**Table 9:** Effects of administration of *C. osmophloeum* leaf extract, nanoemulsion and powder in hydrosol on fasting blood glucose (FBG), oral glucose tolerance test (OGTT), serum insulin and HOMA-IR index in high-fat diet with streptozotocin injection into diabetic rats.

Group	FBG (mg/dL)		OGTT (mg/dL)				Insulin (μg/L)	HOMA-IR
	0 Week	4 Weeks	0 min	30 min	60 min	120 min	4 Weeks	4 Weeks
NC	103.33 ± 9.07 ^Aa^	106.17 ± 7.99 ^Ea^	107.00 ± 7.16 ^Ea^	109.00 ± 7.16 ^Ea^	104.67 ± 7.63 ^Ea^	98.00 ± 5.80 ^Ea^	0.44 ± 0.05 ^C^	2.91 ± 0.31 ^E^
DC	105.50 ± 8.79 ^Ab^	448.00 ± 47.15 ^Aa^	434.33 ± 92.26 ^Ab^	590.50 ± 17.53 ^Aa^	591.83 ± 18.26 ^Aa^	584.83 ± 19.92 ^Aa^	0.90 ± 0.17 ^A^	24.24 ± 8.32 ^A^
HP	103.00 ± 5.35 ^Ab^	352.17 ± 59.54 ^Ba^	359.50 ± 108.80 ^ABc^	394.50 ± 153.62 ^Bb^	444.00 ± 156.16 ^Ba^	404.67 ± 122.86 ^Bb^	0.82 ± 0.13 ^AB^	18.52 ± 7.85 ^AB^
EL	96.67 ± 5.12 ^Ab^	344.5 ± 44.94 ^BCa^	320.33 ± 68.40 ^BCb^	399.50 ± 65.52 ^Bab^	444.17 ± 55.06 ^Ba^	424.50 ± 47.39 ^Ba^	0.77 ± 0.29 ^AB^	14.99 ± 5.52 ^BC^
NL	101.50 ± 9.38 ^Ab^	295.67 ± 46.79 ^Ca^	283.17 ± 66.00 ^BCDc^	326.83 ± 53.31 ^Cbc^	392.17 ± 126.35 ^Ba^	317.17 ± 52.65 ^Cbc^	0.67 ± 0.24 ^ABC^	16.67 ± 6.11 ^CD^
EH	104.50 ± 8.52 ^Ab^	236.67 ± 33.67 ^Da^	244.33 ± 46.84 ^CDb^	300.50 ± 68.74 ^Ca^	311.17 ± 38.88 ^Ca^	264.67 ± 46.72 ^CDb^	0.61 ± 0.34 ^BC^	9.05 ± 4.86 ^CDE^
NH	101.17 ± 2.27 ^Ab^	205.17 ± 22.57 ^Da^	202.83 ± 22.67 ^Db^	241.83 ± 38.55 ^Da^	256.50 ± 42.96 ^Da^	201.83 ± 26.67 ^Db^	0.47 ± 0.06 ^C^	5.83 ± 0.94 ^DE^

Data are presented as mean ± standard deviation (n = 8). Values with different capital letters (A–E) in the same column and small letters (a–c) in the same row for FBG or OGTT are significantly different at *p* < 0.05. HOMA-IR, homeostatic model assessment of insulin resistance; NC, normal control group; DC, high-fat diet with streptozotocin injection at a dose of 65 mg/kg bw; HP, high-fat diet with streptozotocin injection at a dose of 65 mg/kg bw and administration of powder in hydrosols at a dose of 10 mL/kg bw; EL, high-fat diet with streptozotocin injection at a dose of 65 mg/kg bw and administration of leaf extract at a dose of cinnamaldehyde 20 mg/kg bw; NL, high-fat diet with streptozotocin injection at a dose of 65 mg/kg bw and administration of nanoemulsion at a dose of cinnamaldehyde 20 mg/kg bw; EH, high-fat diet with streptozotocin injection at a dose of 65 mg/kg bw and administration of leaf extract at a dose of cinnamaldehyde 60 mg/kg bw; NH, high-fat diet with streptozotocin injection at a dose of 65 mg/kg bw and administration of nanoemulsion at a dose of cinnamaldehyde 60 mg/kg bw.

## References

[1] Huang Y.-C., Chen B.-H. (2023). A Comparative Study on Improving Streptozotocin-Induced Type 2 Diabetes in Rats by Hydrosol, Extract and Nanoemulsion Prepared from Cinnamon Leaves. Antioxidants.

