# Supplementary material for: A Comparative Study on Improving Streptozotocin-Induced Type 2 Diabetes in Rats by Hydrosol, Extract and Nanoemulsion Prepared from Cinnamon Leaves
Source: Antioxidants (Basel). 2022 Dec 23;12(1):29. doi: 10.3390/antiox12010029 (PMC9855112; doi:10.3390/antiox12010029)
Supplement: Supplementary file 1 [file antioxidants-12-00029-s001.zip › antioxidants-2044988-supplementary.pdf]

Supplementary Material
for

# A Comparative Study on Improving Streptozotocin-Induced Type 2 Diabetes in Rats by Hydrosol, Extract and Nanoemulsion Prepared from Cinnamon Leaves

**Yu-Chi Huang [1] and Bing-Huei Chen [1,2,*]**

[1] Department of Food Science, Fu Jen Catholic University, New Taipei City 24205, Taiwan
[2] Department of Nutrition, China Medical University, Taichung 40402, Taiwan
* Correspondence: 002622@mail.fju.edu.tw; Tel.: +886-2-29053626

---

## Materials

*Reagents*

Standards of compounds including neochlorogenic acid (5-O-caffeoylquinic acid) and hyperoside (quercetin-3-O-galactoside) were procured from Chendu Biopurity Phytochemicals (Chengdu, China), while 4-hydroxybenzoic acid, trans-cinnamic acid, cinnamaldehyde and caffeic acid were from Chem Service Inc. (West Chester, PA, USA). Rutin (quercetin-3-O-rutinoside), quercetin, isoquercetin (quercetin-3-O-glucoside), kaempferol, kaempferol-3-β-D-glucopyranoside, p-coumaric acid and eugenol were purchased from Sigma-Aldrich (St. Louis, MO, USA), while coumarin was from Accustandard Co. (St. New Heven, CT, USA).

The HPLC-grade solvents including acetone, methanol and ethanol were obtained from Merck Co. (Darmstadt, Germany), while 99% ethanol and dimethyl sulfoxide (DMSO) were from Sigma-Aldrich. Glacial acetic acid (99.7%) was from Thermo Fisher Scientific (San Jose, CA, USA) and deionized water was made using a Milli-Q water purification system from Millipore Co. (Bedford, MA, USA). Tween 80 was purchased from Yu-Pa Co. (Taipei, Taiwan), while soybean oil was from a local supermarket (Taipei, Taiwan). Both PEG 400 and potassium dihydrogen phosphate were from Sigma-Aldrich.

*Instrumentation*

The ultra-performance LC system (UPLC) coupled with a TQS triple quadrupole MS/MS system (ACQUITY) was from Waters (Milford, MA, USA). The ultrasonicator (model DC400H) was from Hua-Hsiah Scientific Co. (Taipei, Taiwan), while the centrifuge (5810R) was from Eppendorf Co. (Hauppauge, NY, USA). The microcentrifuge (Heraeus Fresco 21) was from Thermo Fisher Scientific Co. (San Jose, CA, USA). The nanoparticle size and zeta potential analyzer (SZ-100) was from Horiba Scientific Co. (Kyoto, Japan). The dynamic light scattering instrument (90 Plus Particle Size Analyzer) was from Brookhaven Instruments Co. (Holtsville, NY, USA). The transmission electron microscope (TEM), model JEM-1400, was from JEOL Co. (Tokyo, Japan). The rotary evaporator (N1200A) was from Eyela Co. (Tokyo, Japan). The low-temperature circulation water tank (B402L) was from Firstek Co. (Taipei, Taiwan).

**Table S1**. The linear regression equation and coefficient of determination ($R^2$) obtained from the calibration curves prepared for 15 standard bioactive compounds.[a]

| **Compound** | **Linear range (ng/g)** | **Linear regression equation** | **$R^2$** |
|---|---|---|---|
| Quercetin | 10-500 | y=36.839x+210.85 | 0.9944 |
| Coumarin | 10-500 | y=1714.5x+120303 | 0.9893 |
| Quercetin-3-*O*-galactoside | 10-500 | y=722.88x+4522.1 | 0.9950 |
| Quercetin-3-*O*-glucoside | 10-500 | y=722.88x+4522.1 | 0.9950 |
| Rutin | 10-500 | y=668.31x-650.36 | 0.9983 |
| Caffeic acid | 10-500 | y=727.73x+1531.9 | 0.9968 |
| Benzoic acid | 10-500 | y=114.77x+10532 | 0.9969 |
| 5-*O*-Caffeoylquinic acid | 10-500 | y=106.26x-314.69 | 0.9930 |
| Cinnamyl alcohol | 10-500 | y=215.24x+3493.4 | 0.9964 |
| *p*-Coumaric acid | 10-500 | y=1061.6x+54869 | 0.9931 |
| Kaempferol 3-β-D-glucopyranoside | 10-500 | y=656.66x+625.15 | 0.9987 |
| Eugenol | 10-500 | y=40.686x+178.22 | 0.9979 |
| Kaempferol | 10-500 | y=3008.5x+7850 | 0.9998 |
| Cinnamaldehyde | 10-500 | y=165.25x-211.54 | 0.9969 |
| *trans*-Cinnamic acid | 10-500 | y=283.46x+2120.5 | 0.9966 |

[a]Calibration curves were prepared from the mean of triplicate analyses of each standard concentration by UPLC-MS/MS.
